# PrIC3: Property Directed Reachability for MDPs

**DOI:** 10.1007/978-3-030-53291-8_27

**Published:** 2020-06-16

**Authors:** Kevin Batz, Sebastian Junges, Benjamin Lucien Kaminski, Joost-Pieter Katoen, Christoph Matheja, Philipp Schröer

**Affiliations:** 8 Microsoft Research Lab, Redmond, WA USA; 9 University of Southern California, Los Angeles, CA USA; 10 RWTH Aachen University, Aachen, Germany; 11 University of California, Berkeley, USA; 12 University College London, London, UK; 13 ETH Zürich, Zürich, Switzerland

## Abstract

IC3 has been a leap forward in symbolic model checking. This paper proposes PrIC3 (pronounced pricy-three), a conservative extension of IC3 to symbolic model checking of MDPs. Our main focus is to develop the theory underlying PrIC3. Alongside, we present a first implementation of PrIC3 including the key ingredients from IC3 such as generalization, repushing, and propagation.

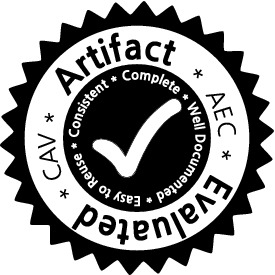



## Introduction

IC3. Also known as property-directed reachability (PDR) [23], IC3 [13] is a symbolic approach for verifying finite transition systems (TSs) against safety properties like “*bad states are unreachable*”. It combines bounded model checking (BMC) [12] and inductive invariant generation. Put shortly, IC3 either proves that a set *B* of bad states is *un*reachable by finding a set of non-*B* states closed under reachability—called an *inductive invariant*—or refutes reachability of *B* by a *counterexample* path reaching *B*. Rather than unrolling the transition relation (as in BMC), IC3 attempts to incrementally strengthen the invariant “no state in *B* is reachable” into an inductive one. In addition, it applies aggressive abstraction to the explored state space, so-called generalization [36]. These aspects together with the enormous advances in modern SAT solvers have led to IC3 ’s success. IC3 has been extended [27, 38] and adapted to software verification [19, 44]. This paper develops a *quantitative* IC3 framework for probabilistic models.

*MDPs.* Markov decision processes (MDPs) extend TSs with discrete probabilistic choices. They are central in planning, AI as well as in modeling randomized distributed algorithms. A key question in verifying MDPs is *quantitative* reachability: “*is the (maximal) probability to reach* *B* *at most* $\lambda$*?*”. Quantitative reachability [5, 6] reduces to solving linear programs (LPs). Various tools support MDP model checking, e.g., Prism [43], Storm [22], modest [34], and EPMC [31]. The LPs are mostly solved using (variants of) value iteration [8, 28, 35, 51]. Symbolic BDD-based MDP model checking originated two decades ago [4] and is rather successful.

*Towards* IC3  *for MDPs.* Despite the success of BDD-based symbolic methods in tools like Prism, IC3 has not penetrated probabilistic model checking yet. The success of IC3 and the importance of quantitative reachability in probabilistic model checking raises the question *whether and how* IC3 * can be adapted—not just utilized—to reason about quantitative reachability in MDPs*. This paper addresses the challenges of answering this question. It extends IC3 in several dimensions to overcome these hurdles, making PrIC3—to our knowledge—*the first* IC3 * framework for quantitative reachability in MDPs*^1^. Notably, PrIC3 is conservative: For a threshold $\lambda = 0$, PrIC3 solves the same qualitative problem *and behaves (almost) the same as standard* IC3. Our main contribution is developing the theory underlying PrIC3, which is accompanied by a proof-of-concept implementation.

### Challenge 1

*(Leaving the Boolean domain).*IC3 iteratively computes *frames*, which are over-approximations of sets of states that can reach *B* in a bounded number of steps. For MDPs, Boolean reachability becomes a *quantitative reachability probability*. This requires a shift: frames become real-valued functions rather than sets of states. Thus, there are infinitely many possible frames—even for finite-state MDPs—just as for infinite-state software [19, 44] and hybrid systems [54]. Additionally, whereas in TSs a state reachable within *k* steps remains reachable on increasing *k*, the reachability probability in MDPs may increase. This complicates ensuring termination of an IC3 algorithm for MDPs.   $\triangle$

### Challenge 2

*(Counterexamples* $\ne$ *single paths).* For TSs, a single cycle-free path^2^ to *B* suffices to refute that “*B* *is not reachable*”. This is not true in the probabilistic setting [32]. Instead, proving that the probability of reaching *B* exceeds the threshold $\lambda$ requires *a set of possibly cyclic paths*—e.g., represented as a sub-MDP [15]—whose probability mass exceeds $\lambda$. Handling sets of paths as counterexamples in the context of IC3 is new.   $\triangle$

### Challenge 3

*(Strengthening).* This key IC3 technique intuitively turns a proof obligation of type (i) “state *s* is unreachable from the initial state $s_I$” into type (ii) “*s*’s *predecessors* are unreachable from $s_I$”. A first issue is that in the quantitative setting, the standard characterization of reachability probabilities in MDPs (the Bellman equations) inherently *reverses* the direction of reasoning (cf. “reverse” IC3 [53]): Hence, strengthening turns (i) “*s* cannot reach $B$” into (ii) “*s*’s *successors* cannot reach $B$”.

A much more challenging issue, however, is that in the quantitative setting obligations of type (i) read “*s* is reachable *with at most probability* $\delta$”. However, the strengthened type (ii) obligation must then read: “*the weighted sum over the reachability probabilities of the successors of* *s* is at most $\delta$”. In general, there are infinitely many possible choices of subobligations for the successors of *s* in order to satisfy the original obligation, because—grossly simplified—there are infinitely many possibilities for *a* and *b* to satisfy weighted sums such as $\tfrac{1}{3} a + \tfrac{2}{3} b \le \delta$. While we only need one choice of subobligations, picking a *good* one is approximately as hard as solving the entire problem altogether. We hence require a heuristic, which is guided by a *user-provided oracle*.   $\triangle$

### Challenge 4

*(Generalization).* “One of the key components of IC3 is [inductive] generalization” [13]. Generalization [36] abstracts single states. It makes IC3 scale, but is *not* essential for correctness. To facilitate generalization, systems should be encoded symbolically, i.e., integer-valued program variables describe states. Frames thus map variables to probabilities. A first aspect is how to effectively present them to an SMT-solver. Conceptually, we use uninterpreted functions and universal quantifiers (encoding program behavior) together with linear real arithmetic to encode the weighted sums occurring when reasoning about probabilities. A second aspect is more fundamental: Abstractly, IC3 ’s generalization guesses an unreachable set of states. We, however, need to guess this set *and* a probability for each state. To be effective, these guesses should moreover eventually yield an inductive frame, which is often highly nonlinear. We propose three SMT-guided interpolation variants for guessing these maps.   $\triangle$

*Structure of this Paper.* We develop PrIC3 gradually: We explain the underlying rationale in Sect. 3. We also describe the core of PrIC3—called $\mathsf{PrIC3} _{\mathcal {H}}$—which resembles closely the main loop of standard IC3, but uses adapted frames and termination criteria (Challenge 1). In line with Challenge 3, $\mathsf{PrIC3} _{\mathcal {H}}$ is parameterized by a heuristic $\mathcal {H}$ which is applied whenever we need to select one out of infinitely many probabilities. No requirements on the quality of $\mathcal {H}$ are imposed. $\mathsf{PrIC3} _{\mathcal {H}}$ is *sound* and always terminates: If it returns ${\texttt {true}}$, then the maximal reachability probability is bounded by $\lambda$. Without additional assumptions about $\mathcal {H}$, $\mathsf{PrIC3} _{\mathcal {H}}$ is *incomplete*: on returning ${\texttt {false}}$, it is unknown whether the returned subMDP is indeed a counterexample (Challenge 2). Section 4 details strengthening (Challenge 3). Section 5 presents a sound *and* complete algorithm $\mathsf{\mathsf{PrIC3}}$ on top of $\mathsf{PrIC3} _{\mathcal {H}}$. Section 6 presents a prototype, discusses our chosen heuristics, and addresses Challenge 4. Section 7 shows some encouraging experiments, but also illustrates need for further progress.

**Related Work.** Just like IC3 has been a symbiosis of different approaches, PrIC3 has been inspired by several existing techniques from the verification of probabilistic systems.

*BMC.* Adaptions of BMC to Markov chains (MCs) with a dedicated treatment of cycles have been pursued in [57]. The encoding in [24] annotates sub-formulae with probabilities. The integrated SAT solving process implicitly unrolls all paths leading to an exponential blow-up. In [52], this is circumvented by grouping paths, discretizing them, and using an encoding with quantifiers and bit-vectors, but without numerical values. Recently, [56] extends this idea to a PAC algorithm by purely propositional encodings and (approximate) model counting [17]. These approaches focus on MCs and are not mature yet.

*Invariant Synthesis.* Quantitative loop invariants are key in analyzing *probabilistic programs* whose operational semantics are (possibly infinite) MDPs [26]. A quantitative invariant *I* maps states to probabilities. *I* is shown to be an invariant by comparing *I* to the result of applying the MDP’s Bellman operator to *I*. Existing approaches for invariant synthesis are, e.g., based on weakest pre-expectations [33, 39, 40, 42, 46], template-based constraint solving [25], notions of martingales [3, 9, 16, 55], and solving recurrence relations [10]. All but the last technique require user guidance.

*Abstraction.* To combat state-space explosion, abstraction is often employed. CEGAR for MDPs [37] deals with explicit sets of paths as counterexamples. Game-based abstraction [30, 41] and partial exploration [14] exploit that not all paths have to be explored to prove bounds on reachability probabilities.

*Statistical Methods and (deep) Reinforcement Learning.* Finally, an avenue that avoids storing a (complete) model are simulation-based approaches (statistical model checking [2]) and variants of reinforcement learning, possibly with neural networks. For MDPs, these approaches yield weak statistical guarantees [20], but may provide good oracles.

## Problem Statement

Our aim is to prove that the *maximal probability* of *reaching* a *set* $B$ *of bad states* from the initial state $s_I$ of a *Markov decision process* $\mathfrak {M}$ is at most some *threshold* $\lambda$. Below, we give a formal description of our problem. We refer to [7, 50] for a thorough introduction.

### Definition 1

**(MDPs).** A *Markov decision process* (*MDP*) is a tuple $\mathfrak {M}= \left( S, \, s_I, \, \mathrm {Act}, \, P\right)$, where *S* is a finite set of *states*, $s_I\in S$ is the *initial state*, $\mathrm {Act}$ is a finite set of *actions*, and $P :S \times \mathrm {Act}\times S \rightarrow [0,1]$ is a *transition probability function*. For state *s*, let $\mathrm {Act}\left( s \right) = \left\{ a \in \mathrm {Act}~\mid ~ \exists s' \in S :P(s,a,s') > 0\right\}$ be the *enabled actions* at *s*. For all states $s \in S$, we require $|\mathrm {Act}\left( s \right) | \ge 1$ and $\sum _{s'\in S} P(s,a,s') = 1$.    $\triangle$

For this paper, we fix an MDP $\mathfrak {M}= \left( S, \, s_I, \, \mathrm {Act}, \, P\right)$, a set of *bad states* $B\subseteq S$, and a threshold $\lambda \in [0, 1]$. The *maximal*^3^ *(unbounded) reachability probability* to eventually reach a state in $B$ from a state *s* is denoted by $\text {Pr}^{\text {max}} \left( s \models \lozenge B \right)$. We characterize $\text {Pr}^{\text {max}} \left( s \models \lozenge B \right)$ using the so-called *Bellman operator*. Let $M^N$ denote the set of functions from *N* to *M*. Anticipating IC3 terminology, we call a function $F \in [0,1]^{S}$ a *frame*. We denote by *F*[*s*] the evaluation of frame *F* for state *s*.

### Definition 2

**(Bellman Operator).** For a set of actions $A \subseteq \mathrm {Act}$, we define the *Bellman operator for* *A* as a frame transformer $\Phi _{A} :[0,1]^{S} \rightarrow [0,1]^{S}$ with

$$\begin{aligned} \Phi _{A} \left( F \right) [s] ~{}={}~{\left\{ \begin{array}{ll} 1, &{} ~\text {if}~ s \in B\\ \max \limits _{a \in A}~ \sum \limits _{s' \in S} P(s, a, s') \cdot F[s']~, &{} ~\text {if}~ s \notin B~. \end{array}\right. } \end{aligned}$$

We write $\Phi _{a}$ for $\Phi _{ \{ a \}}$, $\Phi$ for $\Phi _{\mathrm {Act}}$, and call $\Phi$ simply *the Bellman operator*.    $\triangle$

For every state *s*, the maximal reachability probability $\text {Pr}^{\text {max}} \left( s \models \lozenge B \right)$ is then given by the least fixed point of the Bellman operator $\Phi$. That is,

$$\begin{aligned} \forall \, s:\quad \text {Pr}^{\text {max}} \left( s \models \lozenge B \right) ~{}={}~\bigl (\text {lfp}~ \Phi \bigr )[s]~, \end{aligned}$$

where the underlying partial order on frames is a complete lattice with ordering

$$\begin{aligned} F_1 ~{}\le {}~F_2 \qquad {}\text {iff}{}\qquad \forall \, s \in S:\quad F_1[s] ~{}\le {}~F_2[s]~. \end{aligned}$$

In terms of the Bellman operator, our formal problem statement reads as follows: 
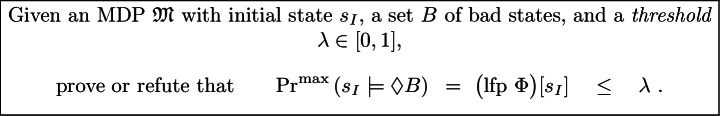
 Whenever $\text {Pr}^{\text {max}} \left( s_I \models \lozenge B \right) \le \lambda$ indeed holds, we say that the MDP $\mathfrak {M}$ is *safe* (with respect to the set of bad states $B$ and threshold $\lambda$); otherwise, we call it *unsafe*.

**Fig. 1.**
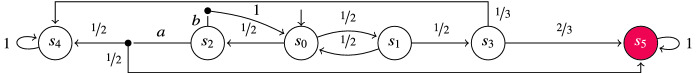
The MDP $\mathfrak {M}$ serving as a running example.

### Recovery Statement 1

For $\lambda = 0$, our problem statement is equivalent to the *qualitative reachability* problem solved by (reverse) standard IC3, i.e, prove or refute that all bad states in $B$ are *unreachable* from the initial state $s_I$.

### Example 1

The MDP $\mathfrak {M}$ in Fig. 1 consists of 6 states with initial state $s_0$ and bad states $B= \{ s_5 \}$. In $s_2$, actions *a* and *b* are enabled; in all other states, one unlabeled action is enabled. We have . Hence, $\mathfrak {M}$ is safe for all thresholds  and unsafe for . In particular, $\mathfrak {M}$ is unsafe for $\lambda = 0$ as $s_5$ is *reachable* from $s_0$.   $\triangle$

## The Core PrIC3 Algorithm

The purpose of PrIC3 is to prove or refute that the maximal probability to reach a bad state in $B$ from the initial state $s_I$ of the MDP $\mathfrak {M}$ is at most $\lambda$. In this section, we explain the rationale underlying PrIC3. Moreover, we describe the core of PrIC3—called $\mathsf{PrIC3} _{\mathcal {H}}$—which bears close resemblance to the main loop of standard IC3 for TSs.

Because of the inherent direction of the Bellman operator, we build PrIC3 on *reverse* IC3 [53], cf. Challenge 3. Reversing constitutes a shift from reasoning along the direction *initial-to-bad* to *bad-to-initial*. While this shift is mostly *inessential* to the fundamentals underlying IC3, the reverse direction is unswayable in the probabilistic setting. Whenever we draw a connection to standard IC3, we thus generally mean *reverse* IC3.

### Inductive Frames

IC3 for TSs operates on (*quali*tative) frames representing sets of states of the TS at hand. A frame *F* can hence be thought of as a mapping^4^ from states to $\{0, 1\}$. In PrIC3 for MDPs, we need to move from a Boolean to a quantitative regime. Hence, a (*quanti*tative) frame is a mapping from states to probabilities in [0, 1].

For a given TS, consider the frame transformer *T* that adds to a given input frame $F'$ all bad states in $B$ and all predecessors of the states contained in $F'$. The rationale of standard (reverse) IC3 is to find a frame $F \in \{0,1\}^{S}$ such that (i) the initial state $s_I$ does not belong to *F* and (ii) applying *T* takes us down in the partial order on frames, i.e.,

$$\begin{aligned} (\textsc {i})\quad F[s_I] ~{}={}~0 \qquad {}\text {and}{}\qquad (\textsc {ii})\quad T(F) ~{}\le {}~F~. \end{aligned}$$

Intuitively, (i) postulates the *hypothesis* that $s_I$ cannot reach $B$ and (ii) expresses that *F* is closed under adding bad states and taking predecessors, thus affirming the hypothesis.

Analogously, the rationale of PrIC3 is to find a frame $F \in [0,1]^{S}$ such that (i) *F* postulates that the probability of $s_I$ to reach $B$ is at most the threshold $\lambda$ and (ii) applying the Bellman operator $\Phi$ to *F* takes us down in the partial order on frames, i.e.,

$$\begin{aligned} (\textsc {i})\quad F[s_I] ~{}\le {}~\lambda \qquad {}\text {and}{}\qquad (\textsc {ii})\quad \Phi (F) ~{}\le {}~F~. \end{aligned}$$

Frames satisfying the above conditions are called *inductive invariants* in IC3. We adopt this terminology. By *Park’s Lemma* [48], which in our setting reads

$$\begin{aligned} \Phi (F) ~{}\le {}~F \quad {}\text {implies}{}\quad \text {lfp}~\Phi ~{}\le {}~F~, \end{aligned}$$

an inductive invariant *F* would indeed *witness* that $\text {Pr}^{\text {max}} \left( s_I \models \lozenge B \right) \le \lambda$, because

$$\begin{aligned} \text {Pr}^{\text {max}} \left( s_I \models \lozenge B \right) ~{}={}~\bigl ( \text {lfp}~ \Phi \bigr )[s_I] ~{}\le {}~F[s_I] ~{}\le {}~\lambda ~. \end{aligned}$$

If no inductive invariant exists, then standard IC3 will find a counterexample: a *path* from the initial state $s_I$ to a bad state in $B$, which serves as a witness to refute. Analogously, PrIC3 will find a counterexample, but of a different kind: Since single paths are insufficient as counterexamples in the probabilistic realm (Challenge 2), PrIC3 will instead find a *subsystem* of states of the MDP witnessing $\text {Pr}^{\text {max}} \left( s_I \models \lozenge B \right) > \lambda$.

### The PrIC3 Invariants

Analogously to standard IC3, PrIC3 aims to find the inductive invariant by maintaining a *sequence of frames* $F_0 \le F_1 \le F_2 \le \ldots$ such that $F_i[s]$ overapproximates the maximal probability of reaching *B* from *s* within *at most i steps*. This *i-step-bounded reachability probability* $\text {Pr}^{\text {max}} \left( s \models \lozenge ^{\le i} B \right)$ can be characterized using the Bellman operator: $\Phi \left( \mathbf {0} \right)$ is the 0-step probability; it is 1 for every $s \in B$ and 0 otherwise. For any $i \ge 0$, we havewhere $\mathbf {0}$, the frame that maps every state to 0, is the least frame of the underlying complete lattice. For a finite MDP, the *unbounded* reachability probability is then given by the limitwhere $(*)$ is a consequence of the well-known Kleene fixed point theorem [45].

The sequence $F_0 \le F_1 \le F_2 \le \ldots$ maintained by PrIC3 should frame-wise overapproximate the increasing sequence $\Phi \left( \mathbf {0} \right) \le \Phi ^{2} \left( \mathbf {0} \right) \le \Phi ^{3} \left( \mathbf {0} \right) \ldots$. Pictorially: 
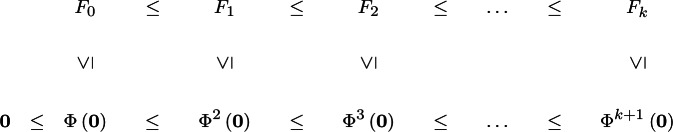
 However, the sequence $\Phi \left( \mathbf {0} \right) ,\, \Phi ^{2} \left( \mathbf {0} \right) ,\, \Phi ^{3} \left( \mathbf {0} \right) ,\, \ldots$ will never explicitly be known to PrIC3. Instead, PrIC3 will ensure the above frame-wise overapproximation property implicitly by enforcing the so-called PrIC3 * invariants* on the frame sequence $F_0,\, F_1,\, F_2,\, \ldots$. Apart from allowing for a threshold $0 \le \lambda \le 1$ on the maximal reachability probability, these invariants coincide with the standard IC3 invariants (where $\lambda = 0$ is fixed). Formally:

#### Definition 3

**(** **Invariants).** Frames $F_0,\, \ldots ,\, F_k$, for $k \ge 0$, satisfy the PrIC3 * invariants*, a fact we will denote by $\textsf {{PrIC3Inv}} \left( F_0,\, \ldots ,\, F_k \right)$, if all of the following hold:

$$\begin{array}{l@{\qquad }l} {\mathbf {1.}}\quad {\mathbf {Initiality:}} &{} F_0 ~{}={}~\Phi \left( \mathbf {0} \right) \\ {\mathbf {2.}}\quad {\mathbf {Chain \ Property:}} &{} \forall \, 0 \le i< k :\quad F_i ~{}\le {}~F_{i+1} \\ {\mathbf {3.}}\quad {\mathbf {Frame}{} \mathbf - \mathbf {safety:}} &{} \forall \, 0 \le i \le k :\quad F_i[s_I] ~{}\le {}~\lambda \\ {\mathbf {4.}}\quad {\mathbf {Relative \ Inductivity:}} &{} \forall \, 0 \le i < k :\quad \Phi \left( F_i \right) ~{}\le {}~F_{i+1} \end{array}$$

   $$\triangle $$

The PrIC3 invariants enforce the above picture: The *chain property* ensures $F_0 \le F_1 \le \ldots \le F_k$. We have $\Phi \left( \mathbf {0} \right) = F_0 \le F_0$ by *initiality*. Assuming $\Phi ^{i+1} \left( \mathbf {0} \right) \le F_i$ as induction hypothesis, monotonicity of $\Phi$ and *relative inductivity* imply $\Phi ^{i+2} \left( \mathbf {0} \right) \le \Phi (F_i) \le F_{i+1}$.

By overapproximating $\Phi \left( \mathbf {0} \right) ,\, \Phi ^{2} \left( \mathbf {0} \right) ,\, \ldots ,\, \Phi ^{k+1} \left( \mathbf {0} \right)$, the frames $F_0,\,\ldots ,\, F_k$ in effect bound the maximal step-bounded reachability probability of every state:

#### Lemma 1

Let frames $F_0,\, \ldots ,\, F_k$ satisfy the PrIC3 invariants. Then

$$\begin{aligned} \forall \, s~~ \forall \, i \le k:\quad \text {Pr}^{\text {max}} \left( s \models \lozenge ^{\le i} B \right) ~{}\le {}~F_i[s]. \end{aligned}$$

In particular, Lemma 1 together with *frame-safety* ensures that the maximal step-bounded reachability probability of the *initial state* $s_I$ to reach $B$ is at most the threshold $\lambda$.

As for proving that the *unbounded* reachability probability is also at most $\lambda$, it suffices to find two consecutive frames, say $F_i$ and $F_{i+1}$, that coincide:

#### Lemma 2

Let frames $F_0,\, \ldots ,\, F_k$ satisfy the PrIC3 invariants. Then

$$\begin{aligned} \exists \, i < k :\quad F_i ~{}={}~F_{i+1} \qquad {}\text {implies}{}\qquad \text {Pr}^{\text {max}} \left( s_I \models \lozenge B \right) ~{}\le {}~\lambda ~. \end{aligned}$$

#### Proof

$F_i = F_{i + 1}$ and *relative inductivity* yield $\Phi (F_i) \le F_{i+1} = F_i$, rendering $F_i$ *inductive*. By Park’s lemma (cf. Sect. 3.1), we obtain $\text {lfp}~\Phi \le F_i$ and—by *frame-safety*—conclude

$$\begin{aligned} \text {Pr}^{\text {max}} \left( s_I \models \lozenge B \right) ~{}={}~\bigl ( \text {lfp}~\Phi \bigr ) [s_I] ~{}\le {}~F_i [s_I] ~{}\le {}~\lambda ~. \end{aligned}$$

   $$\square $$

### Operationalizing the PrIC3 Invariants for Proving Safety

Lemma 2 gives us a clear angle of attack for *proving* an MDP safe: Repeatedly add and refine frames approximating step-bounded reachability probabilities for more and more steps while enforcing the PrIC3 invariants (cf. Definition 3.2) until two consecutive frames coincide.
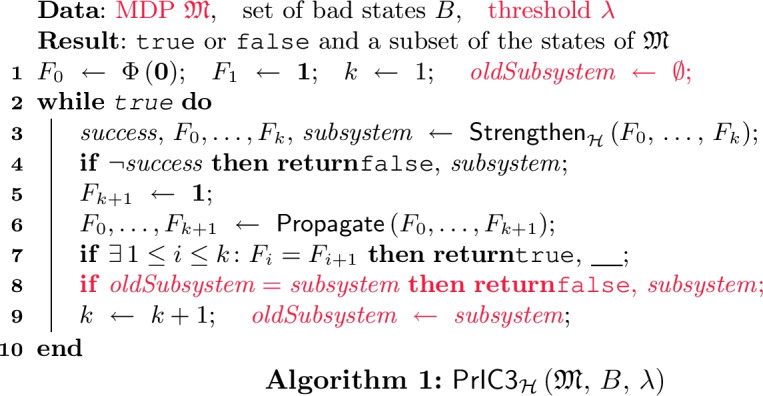
 Analogously to standard IC3, this approach is taken by the core loop $\mathsf{PrIC3} _{\mathcal {H}}$ depicted in Algorithm 1; differences to the main loop of IC3 (cf. [23, Fig. 5]) are highlighted in  . A particular difference is that $\mathsf{PrIC3} _{\mathcal {H}}$ is parameterized by a heuristic $\mathcal {H}$ for finding suitable probabilities (see Challenge 3). Since the precise choice of $\mathcal {H}$ is irrelevant for the soundness of $\mathsf{PrIC3} _{\mathcal {H}}$, we defer a detailed discussion of suitable heuristics to Sect. 4.

As input, $\mathsf{PrIC3} _{\mathcal {H}}$ takes an MDP $\mathfrak {M}= \left( S, \, s_I, \, \mathrm {Act}, \, P\right)$, a set $B\subseteq S$ of bad states, and a threshold $\lambda \in [0,1]$. Since the input is never changed, we assume it to be *globally available*, also to subroutines. As output, $\mathsf{PrIC3} _{\mathcal {H}}$ returns ${\texttt {true}}$ if two consecutive frames become equal. We hence say that $\mathsf{PrIC3} _{\mathcal {H}}$ is *sound* if it only returns ${\texttt {true}}$ if $\mathfrak {M}$ is safe.

We will formalize soundness using Hoare triples. For precondition $\phi$, postcondition $\psi$, and program *P*, the triple $\big \{\, \phi \,\big \} \, P \, \big \{\, \psi \,\big \}$ is *valid* (for partial correctness) if, whenever program *P* starts in a state satisfying precondition $\phi$ and terminates in some state $s'$, then $s'$ satisfies postcondition $\psi$. Soundness of $\mathsf{PrIC3} _{\mathcal {H}}$ then means validity of the tripleLet us briefly go through the individual steps of $\mathsf{PrIC3} _{\mathcal {H}}$ in Algorithm 1 and convince ourselves that it is indeed sound. After that, we discuss why $\mathsf{PrIC3} _{\mathcal {H}}$ terminates and what happens if it is unable to prove safety by finding two equal consecutive frames.

**How** $\varvec{\mathsf{PrIC3} _{\mathcal {H}}}$ **works.** Recall that $\mathsf{PrIC3} _{\mathcal {H}}$ maintains a sequence of frames $F_0,\, \ldots ,\, F_k$ which is initialized in l. 1 with $k = 1$, $F_0 = \Phi \left( \mathbf {0} \right)$, and $F_1 = \mathbf {1}$, where the frame $\mathbf {1}$ maps every state to 1. Every time upon entering the **while**-loop in terms l. 2, the initial segment $F_0,\, \ldots ,\, F_{k-1}$ satisfies all PrIC3 invariants (cf. Definition 3), whereas the full sequence $F_0,\, \ldots ,\, F_k$ potentially violates frame-safety as it is possible that $F_{k}[s_I] > \lambda$.

In l. 3, procedure $\mathsf{Strengthen} _{\mathcal {H}}$—detailed in Sect. 4—is called to restore *all* PrIC3 invariants on the *entire* frame sequence: It either returns ${\texttt {true}}$ if successful or returns ${\texttt {false}}$ and a counterexample (in our case a subsystem of the MDP) if it was unable to do so. To ensure soundness of $\mathsf{PrIC3} _{\mathcal {H}}$, it suffices that $\mathsf{Strengthen} _{\mathcal {H}}$ restores the PrIC3 invariants whenever it returns ${\texttt {true}}$. Formally, $\mathsf{Strengthen} _{\mathcal {H}}$ must meet the following specification:

#### Definition 4

Procedure $\mathsf{Strengthen} _{\mathcal {H}}$ is *sound* if the following Hoare triple is valid:

$$\begin{aligned}&\big \{\, \textsf {{PrIC3Inv}} \left( F_0,\ldots ,F_{k-1} \right) ~{}\wedge {}~F_{k-1} \le F_k ~{}\wedge {}~\Phi \left( F_{k-1} \right) \le F_k \,\big \}\\&\qquad \textit{success}, \, F_0, \ldots ,F_k, \, \underline{}~{}\leftarrow {}~\mathsf{Strengthen} _{\mathcal {H}}\left( F_0,\, \ldots ,\, F_k \right) \\&\big \{\, \textit{success}~{}\Rightarrow {}~\textsf {{PrIC3Inv}} \left( F_0, \ldots , F_k \right) \,\big \}. \end{aligned}$$

If $\mathsf{Strengthen} _{\mathcal {H}}$ returns ${\texttt {true}}$, then a new frame $F_{k+1} = \mathbf {1}$ is created in l. 5. After that, the (now initial) segment $F_0,\, \ldots ,\, F_k$ again satisfies all PrIC3 invariants, whereas the full sequence $F_0,\, \ldots ,\, F_{k+1}$ potentially violates frame-safety at $F_{k+1}$. *Propagation* (l. 6) aims to speed up termination by updating $F_{i+1}[s]$ by $F_{i}[s]$ iff this does not violate relative inductivity. Consequently, the previously mentioned properties remain unchanged.

If $\mathsf{Strengthen} _{\mathcal {H}}$ returns ${\texttt {false}}$, the PrIC3 invariants—premises to Lemma 2 for witnessing safety—cannot be restored and $\mathsf{PrIC3} _{\mathcal {H}}$ terminates returning ${\texttt {false}}$ (l. 4). Returning ${\texttt {false}}$ (also possible in l. 8) has by specification no affect on soundness of $\mathsf{PrIC3} _{\mathcal {H}}$.

In l. 7, we check whether there exist two identical consecutive frames. If so, Lemma 2 yields that the MDP is safe; consequently, $\mathsf{PrIC3} _{\mathcal {H}}$ returns ${\texttt {true}}$. Otherwise, we increment *k* and are in the same setting as upon entering the loop, now with an increased frame sequence; $\mathsf{PrIC3} _{\mathcal {H}}$ then performs another iteration. In summary, we obtain:

#### Theorem 1

**(Soundness of** **).** If $\mathsf{Strengthen} _{\mathcal {H}}$ is sound and $\mathsf{Propagate}$ does not affect the PrIC3 invariants, then $\mathsf{PrIC3} _{\mathcal {H}}$ is sound, i.e., the following triple is valid:


$\varvec{\mathsf{PrIC3} _{\mathcal {H}}}$ **Terminates for Unsafe MDPs.** If the MDP is unsafe, then there exists a step-bound *n*, such that $\text {Pr}^{\text {max}} \left( s_I \models \lozenge ^{\le n} B \right) > \lambda$. Furthermore, any sound implementation of $\mathsf{Strengthen} _{\mathcal {H}}$ (cf. Definition 4) either immediately terminates $\mathsf{PrIC3} _{\mathcal {H}}$ by returning ${\texttt {false}}$ or restores the PrIC3 invariants for $F_0,\, \ldots ,\, F_k$. If the former case never arises, then $\mathsf{Strengthen} _{\mathcal {H}}$ will eventually restore the PrIC3 invariants for a frame sequence of length $k = n$. By Lemma 1, we have $F_n[s_I] \ge \text {Pr}^{\text {max}} \left( s_I \models \lozenge ^{\le n} B \right) > \lambda$ contradicting frame-safety.

$\varvec{\mathsf{PrIC3} _{\mathcal {H}}}$ **Terminates for Safe MDPs.** Standard IC3 terminates on safe finite TSs as there are only finitely many different frames, making every ascending chain of frames eventually stabilize. For us, frames map states to probabilities (Challenge 1), yielding *infinitely many possible frames* even for finite MDPs. Hence, $\mathsf{Strengthen} _{\mathcal {H}}$ need not ever yield a stabilizing chain of frames. If it continuously fails to stabilize while repeatedly reasoning about the same set of states, we give up. $\mathsf{PrIC3} _{\mathcal {H}}$ checks this by comparing the subsystem $\mathsf{Strengthen} _{\mathcal {H}}$ operates on with the one it operated on in the previous loop iteration (l. 8).

#### Theorem 2

If $\mathsf{Strengthen} _{\mathcal {H}}$ and Propagate terminate, then $\mathsf{PrIC3} _{\mathcal {H}}$ terminates.

#### Recovery Statement 2

For qual. reachability ($\lambda =0$), $\mathsf{PrIC3} _{\mathcal {H}}$ never terminates in l. 8.

$\varvec{\mathsf{PrIC3} _{\mathcal {H}}}$ **is Incomplete.** Standard IC3 either proves safety or returns ${\texttt {false}}$ and a counterexample—a single path from the initial to a bad state. As single paths are insufficient as counterexamples in MDPs (Challenge 2), $\mathsf{PrIC3} _{\mathcal {H}}$ instead returns a *subsystem* of the MDP $\mathfrak {M}$ provided by $\mathsf{Strengthen} _{\mathcal {H}}$. However, as argued above, we cannot trust $\mathsf{Strengthen} _{\mathcal {H}}$ to provide a stabilizing chain of frames. Reporting ${\texttt {false}}$ thus only means that the given MDP *may* be unsafe; the returned subsystem has to be analyzed further.

The full PrIC3 algorithm presented in Sect. 5 addresses this issue. Exploiting the subsystem returned by $\mathsf{PrIC3} _{\mathcal {H}}$, PrIC3 returns ${\texttt {true}}$ if the MDP is safe; otherwise, it returns ${\texttt {false}}$ and provides a true counterexample witnessing that the MDP is unsafe.

#### Example 2

**Fig. 2.**
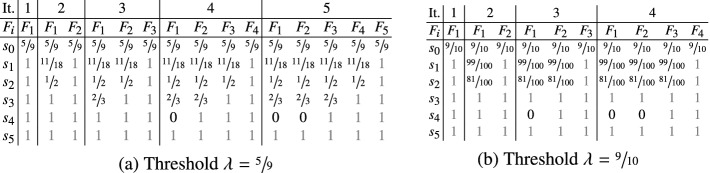
Two runs of $\mathsf{PrIC3} _{\mathcal {H}}$ on the Markov chain induced by selecting action *a* in Fig. 1. For every iteration, frames are recorded after invocation of $\mathsf{Strengthen} _{\mathcal {H}}$.

We conclude this section with two example executions of $\mathsf{PrIC3} _{\mathcal {H}}$ on a simplified version of the MDP in Fig. 1. Assume that action *b* has been removed. Then, for every state, exactly one action is enabled, i.e., we consider a Markov chain. Figure 2 depicts the frame sequences computed by $\mathsf{PrIC3} _{\mathcal {H}}$ (for a reasonable $\mathcal {H}$) on that Markov chain for two thresholds:  and . In particular, notice that *proving the coarser bound of*  *requires fewer frames than proving the exact bound of* .    $\triangle$

## Strengthening in $\mathsf{PrIC3} _{\mathcal {H}}$

When the main loop of $\mathsf{PrIC3} _{\mathcal {H}}$ has created a new frame $F_{k} = \mathbf {1}$ in its previous iteration, this frame may violate frame-safety (Definition 3.3) because of $F_{k}[s_I] = 1 \not \le \lambda$. The task of $\mathsf{Strengthen} _{\mathcal {H}}$ is to restore the PrIC3 invariants on *all* frames $F_0,\ldots ,F_k$. To this end, our first *obligation* is to lower the value in frame $i = k$ for state $s = s_I$ to $\delta = \lambda \in [0,1]$. We denote such an obligation by $(i,s,\delta )$. Observe that implicitly $\delta = 0$ in the qualitative case, i.e., when proving unreachability. An obligation $(i,s,\delta )$ is *resolved* by updating the values assigned to state *s* in *all frames* $F_1, \ldots , F_i$ to at most $\delta$. That is, for all $j \le i$, we set $F_{j}[s]$ to the minimum of $\delta$ and the original value $F_{j}[s]$. Such an update affects neither initiality nor the chain property (Definitions 3.1, 3.2). It may, however, violate relative inductivity (Definition 3.4), i.e., $\Phi \left( F_{i-1} \right) \le F_{i}$. Before resolving obligation $(i,s,\delta )$, we may thus have to further decrease some entries in $F_{i-1}$ as well. Hence, *resolving obligations may spawn additional obligations* which have to be resolved first to maintain relative inductivity. In this section, we present a generic instance of $\mathsf{Strengthen} _{\mathcal {H}}$ meeting its specification (Definition 4) and discuss its correctness.

$\varvec{\mathsf{Strengthen} _{\mathcal {H}}}$ **by Example.** $\mathsf{Strengthen} _{\mathcal {H}}$ is given by the pseudo code in Algorithm 2; differences to standard IC3 (cf. [23, Fig. 6]) are highlighted in  . Intuitively, $\mathsf{Strengthen} _{\mathcal {H}}$ attempts to recursively resolve all obligations until either both frame-safety and relative inductivity are restored for *all* frames or it detects a *potential counterexample* justifying why it is unable to do so. We first consider an execution where the latter does not arise: 
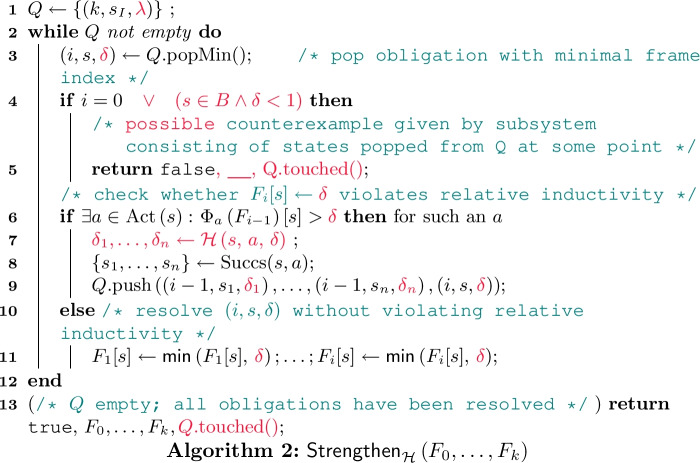


### Example 3

We zoom in on Example 2: Prior to the second iteration, we have created the following three frames assigning values to the states $s_0,s_5$:To keep track of unresolved obligations $(i,s,\delta )$, $\mathsf{Strengthen} _{\mathcal {H}}$ employs a priority queue *Q* which pops obligations with minimal frame index *i* first. Our first step is to ensure frame-safety of $F_2$, i.e., alter $F_2$ so that ; we thus initialize the queue *Q* with the initial obligation  (l. 1). To do so, we check whether updating $F_2[s_0]$ to  would invalidate relative inductivity (l. 6). This is indeed the case:To restore relative inductivity, $\mathsf{Strengthen} _{\mathcal {H}}$ spawns one new obligation for each relevant successor of $s_0$. These have to be resolved before retrying to resolve the old obligation.^5^

*In contrast to standard* IC3 *, spawning obligations involves finding suitable probabilities* $\delta$ (l. 7). In our example this means we have to spawn two obligations $(1,s_1,\delta _1)$ and $(1,s_2,\delta _2)$ such that . There are *infinitely many choices* for $\delta _1$ and $\delta _2$ satisfying this inequality. Assume some heuristic $\mathcal {H}$ chooses  and ; we push obligations , , and  (ll. 8, 9). In the next iteration, we first pop obligation  (l. 3) and find that it can be resolved without violating relative inductivity (l. 6). Hence, we set $F_1[s_1]$ to  (l. 11); no new obligation is spawned. Obligation  is resolved analogously; the updated frame is . Thereafter, our initial obligation  can be resolved; relative inductivity is restored for $F_0,F_1,F_2$. Hence, $\mathsf{Strengthen} _{\mathcal {H}}$ returns ${\texttt {true}}$ together with the updated frames.    $\triangle$

$\varvec{\mathsf{Strengthen} _{\mathcal {H}}}$ **is Sound.** Let us briefly discuss why Algorithm 2 meets the specification of a sound implemenation of $\mathsf{Strengthen} _{\mathcal {H}}$ (Definition 4): First, we observe that Algorithm 2 alters the frames—and thus potentially invalidates the PrIC3 invariants—only in l. 11 by resolving an obligation $(i,s,\delta )$ with $\Phi \left( F_{i-1} \right) [s] \le \delta$ (due to the check in l. 6).

Let $F\left\langle s \mapsto \delta \right\rangle$ denote the frame *F* in which *F*[*s*] is set to $\delta$, i.e.,

$$\begin{aligned} F\left\langle s \mapsto \delta \right\rangle [s'] ~{}={}~{\left\{ \begin{array}{ll} \delta , &{}\text {if}~s' = s, \\ F[s'], &{}\text {otherwise}. \end{array}\right. } \end{aligned}$$

Indeed, resolving obligation $(i,s,\delta )$ in l. 11 lowers the values assigned to state *s* to at most $\delta$ *without* invalidating the PrIC3 invariants:

### Lemma 3

Let $(i,s,\delta )$ be an obligation and $F_0,\ldots ,F_i$, for $i > 0$, be frames with $\Phi \left( F_{i-1} \right) [s] \le \delta$. Then $\textsf {{PrIC3Inv}} \left( F_0, \ldots , F_i \right) \text { implies }$


Crucially, the precondition of Definition 4 guarantees that all PrIC3 invariants except frame safety hold initially. Since these invariants are never invalidated due to Lemma 3, Algorithm 2 is a sound implementation of $\mathsf{Strengthen} _{\mathcal {H}}$ if it restores frame safety whenever it returns ${\texttt {true}}$, i.e., once it leaves the loop with an empty obligation queue *Q* (ll. 12–13). Now, an obligation $(i,s,\delta )$ is only popped from *Q* in l. 3. As $(i,s,\delta )$ is added to *Q* upon reaching l. 9, the size of *Q* can only ever be reduced (without returning ${\texttt {false}}$) by resolving $(i,s,\delta )$ in l. 11. Hence, Algorithm 2 does not return ${\texttt {true}}$ unless it restored frame safety by resolving, amongst all other obligations, the initial obligation $(k,s_I,\lambda )$. Consequently:

### Lemma 4

Procedure $\mathsf{Strengthen} _{\mathcal {H}}$ is sound, i.e., it satisfies the specification in Definition 4.

### Theorem 3

Procedure $\mathsf{PrIC3} _{\mathcal {H}}$ is sound, i.e., satisfies the specification in Theorem 1.

We remark that, analogously to standard IC3, resolving an obligation in l. 11 may be accompanied by *generalization*. That is, we attempt to update the values of multiple states at once. Generalization is, however, highly non-trivial in a probabilistic setting. We discuss three possible approaches to generalization in Sect. 6.2.

$\varvec{\mathsf{Strengthen} _{\mathcal {H}}{}}$ **Terminates.** We now show that $\mathsf{Strengthen} _{\mathcal {H}}$ as in Algorithm 2 terminates. The only scenario in which $\mathsf{Strengthen} _{\mathcal {H}}$ may not terminate is if it keeps spawning obligations in l. 9. Let us thus look closer at how obligations are spawned: Whenever we detect that resolving an obligation $(i,s,\delta )$ would violate relative inductivity for some action *a* (l. 6), we first need to update the values of the successor states $s_1,\ldots , s_n \in \text {Succs}(s,a)$ in frame $i{-}1$, i.e., we push the obligations $(i{-}1,s_1,\delta _1), \ldots , (i{-}1, s_n, \delta _n)$ which have to be resolved first (ll. 7–9). It is noteworthy that, for a TS, a single action leads to a single successor state $s_1$. Algorithm 2 employs a heuristic $\mathcal {H}$ to determine the probabilities required for pushing obligations (l. 7). Assume for an obligation $(i,s,\delta )$ that the check in l. 6 yields $\exists a \in \mathrm {Act}\left( s \right) :\Phi _{a} \left( F_{i-1} \right) [s] > \delta$. Then $\mathcal {H}$ takes *s*, *a*, $\delta$ and reports some probability $\delta _j$ for every *a*-successor $s_j$ of *s*. However, an arbitrary heuristic of type $\mathcal {H} :S \,\times \, \mathrm {Act}\,\times \, [0,1] \rightarrow [0,1]^*$ may lead to non-terminating behavior: If $\delta _1, \ldots , \delta _n = F_{i-1}[s_1], \ldots F_{i-1}[s_n]$, then the heuristic has no effect. It is thus natural to require that an *adequate* heuristic $\mathcal {H}$ yields probabilities such that the check $\Phi _{a} \left( F_{i-1} \right) [s] > \delta$ in l. 6 cannot succeed twice for the *same obligation* $(i,s,\delta )$ and *same action a*. Formally, this is guaranteed by the following:

### Definition 5

Heuristic $\mathcal {H}$ is *adequate* if the following triple is valid (for any frame *F*):   $\triangle$

Details regarding our implementation of heuristic $\mathcal {H}$ are found in Sect. 6.1.

For an adequate heuristic, attempting to resolve an obligation $(i,s,\delta )$ (ll. 3 – 11) either succeeds after spawning it at most $|\mathrm {Act}(s)|$ times or $\mathsf{Strengthen} _{\mathcal {H}}$ returns ${\texttt {false}}$. By a similar argument, attempting to resolve an obligation  leads to at most $\sum _{a\in \mathrm {Act}(s)} |\{ s' \in S \mid P(s, a, s') > 0\}|$ other obligations of the form . Consequently, the total number of obligations spawned by Algorithm 2 is bounded. Since Algorithm 2 terminates if all obligations have been resolved (l. 12) and each of its loop iterations either returns ${\texttt {false}}$, spawns obligations, or resolves an obligation, we conclude:

### Lemma 5

$\mathsf{Strengthen} _{\mathcal {H}}(F_0,\, \ldots ,\, F_k)$ terminates for every adequate heuristic $\mathcal {H}$.

### Recovery Statement 3

Let $\mathcal {H}$ be adequate. Then for qualitative reachability ($\lambda = 0$), all obligations spawned by $\mathsf{Strengthen} _{\mathcal {H}}$ as in Algorithm 2 are of the form (*i*, *s*, 0).

$\varvec{\mathsf{Strengthen} _{\mathcal {H}}{}}$ **returns** **.** There are two cases in which $\mathsf{Strengthen} _{\mathcal {H}}$ fails to restore the PrIC3 invariants and returns ${\texttt {false}}$. The first case (the left disjunct of l. 4) is that we encounter an obligation for frame $F_0$. Resolving such an obligation would inevitably violate *initiality*; analogously to standard IC3, we thus return ${\texttt {false}}$.

The second case (the right disjunct of l. 4) is that we encounter an obligation $(i,s,\delta )$ for a bad state $s \in B$ with a probability $\delta < 1$ (though, obviously, all $s \in B$ have probability ${=}1$). Resolving such an obligation would inevitably prevents us from restoring *relative inductivity*: If we updated $F_i[s]$ to $\delta$, we would have $\Phi \left( F_{i-1} \right) [s] = 1 > \delta = F_{i}[s]$. Notice that, in contrast to standard IC3, this second case *can* occur in PrIC3:

### Example 4

Assume we have to resolve an obligation  for the MDP in Fig. 1. This involves spawning obligations $(i{-}1, s_4,\delta _1)$ and $(i{-}1, s_5, \delta _2)$, where $s_5$ is a bad state, such that . Even for $\delta _1 = 0$, this is only possible if .    $\triangle$

$\varvec{\mathsf{Strengthen} _{\mathcal {H}}{}}$ **Cannot Prove Unsafety.** If standard IC3 returns ${\texttt {false}}$, it proves unsafety by constructing a counterexample, i.e., *a single path from the initial state to a bad state*. If PrIC3 returns ${\texttt {false}}$, there are two possible reasons: *Either* the MDP is indeed unsafe, *or* the heuristic $\mathcal {H}$ at some point selected probabilities in a way such that $\mathsf{Strengthen} _{\mathcal {H}}$ is unable to restore the $\mathsf{PrIC3}$ invariants (even though the MDP might in fact be safe). $\mathsf{Strengthen} _{\mathcal {H}}$ thus only returns a *potential* counterexample which either proves unsafety or indicates that our heuristic was inappropriate.

Counterexamples in our case consist of subsystems rather than a single path (see Challenge 2 and Sect. 5). $\mathsf{Strengthen} _{\mathcal {H}}$ hence returns the set $Q.\text {touched}()$ of all states that eventually appeared in the obligation queue. This set is a conservative approximation, and optimizations as in [1] may be beneficial. Furthermore, in the qualitative case, our potential counterexample subsumes the counterexamples constructed by standard IC3:

### Recovery Statement 4

Let $\mathcal {H} _0$ be the adequate heuristic mapping every state to 0. For qual. reachability ($\lambda = 0$), if $\textit{success}= {\texttt {false}}$ is returned by $\mathsf{Strengthen} _{\mathcal {H} _0}\left( F_0,\, \ldots ,\, F_k \right)$, then $Q.\text {touched}()$ contains a path from the initial to a bad state.^6^

## Dealing with Potential Counterexamples

Recall that our core algorithm $\mathsf{PrIC3} _{\mathcal {H}}$ is incomplete for a fixed heuristic $\mathcal {H}$: It cannot give a conclusive answer whenever it finds a potential counterexample for two possible reasons: Either the heuristic $\mathcal {H}$ turned out to be inappropriate or the MDP is indeed unsafe. The idea to overcome the former is to call $\mathsf{PrIC3} _{\mathcal {H}}$ finitely often in an outer loop that generates new heuristics until we find an appropriate one: If $\mathsf{PrIC3} _{\mathcal {H}}$ still does not report safety of the MDP, then it is indeed unsafe. We do not blindly generate new heuristics, but use the potential counterexamples returned by $\mathsf{PrIC3} _{\mathcal {H}}$ to refine the previous one.
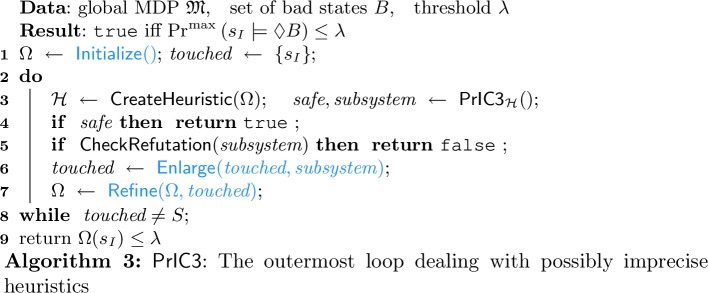


Let consider the procedure $\mathsf{\mathsf{PrIC3}}$ in Algorithm 3 which wraps our core algorithm $\mathsf{PrIC3} _{\mathcal {H}}$ in more detail: First, we create an *oracle* $\Omega :S \rightarrow [0,1]$ which (roughly) *estimates* the probability of reaching $B$ for every state. A *perfect oracle* would yield *precise* maximal reachability probabilites, i.e., $\Omega (s) = \text {Pr}^{\text {max}} \left( s \models \lozenge B \right)$ for every state *s*. We construct oracles by  (highlighted in  ). Examples of implementations of all user-supplied methods in Algorithm 3 are discussed in Sect. 7.

Assuming the oracle is good, but not perfect, we construct an adequate heuristic $\mathcal {H}$ selecting probabilities based on the oracle^7^ for all successors of a given state: There are various options. The simplest is to pass-through the oracle values. A version that is more robust against noise in the oracle is discussed in Sect. 6. We then invoke $\mathsf{PrIC3} _{\mathcal {H}}$. If $\mathsf{PrIC3} _{\mathcal {H}}$ reports safety, the MDP is indeed safe by the soundness of $\mathsf{PrIC3} _{\mathcal {H}}$.

**Check Refutation.** If $\mathsf{PrIC3} _{\mathcal {H}}$ does not report safety, it reports a subsystem that hints to a *potential* counterexample. Formally, this subsystem is a subMDP of states that were ‘visited’ during the invocation of $\mathsf{Strengthen} _{\mathcal {H}}$.

### Definition 6

**(subMDP).** Let $\mathfrak {M}= \left( S, \, s_I, \, \mathrm {Act}, \, P\right)$ be an MDP and let $S' \subseteq S$ with $s_I\in S'$. We call $\mathfrak {M}_{S'} = \left( S', \, s_I, \, \mathrm {Act}, \, P' \right)$ the *subMDP induced by* $\mathfrak {M}$ *and* $S'$, where for all $s,s' \in S'$ and all $a \in \mathrm {Act}$, we have $P' (s, a, s') = P(s, a, s')$.    $\triangle$

A subMDP $\mathfrak {M}_{S'}$ may be substochastic where missing probability mass never reaches a bad state. Definition 1 is thus relaxed: For all states $s \in S'$ we require that $\sum _{s'\in S'} P(s,a,s') \le 1$.If the subsystem is unsafe, we can conclude that the original MDP $\mathfrak {M}$ is also safe.

### Lemma 6

If $\mathfrak {M}'$ is a subMDP of $\mathfrak {M}$ and $\mathfrak {M}'$ is unsafe, then $\mathfrak {M}$ is also unsafe.

The role of $\mathsf{CheckRefutation}$ is to establish whether the subsystem is indeed a true counterexample or a spurious one. Formally, $\mathsf{CheckRefutation}$ should ensure:

$$\begin{aligned} \big \{\, {\texttt {true}} \,\big \} \, \textit{res} \leftarrow \mathsf{CheckRefutation} \left( \textit{subsystem}\right) \, \big \{\, \textit{res} = {\texttt {true}}~{}\Leftrightarrow {}~ \mathfrak {M}_{\textit{subsystem}} \text { unsafe} \,\big \} . \end{aligned}$$

Again, $\mathsf{\mathsf{PrIC3}}$ is backward compatible in the sense that a single fixed heuristic is always sufficient when reasoning about reachability ($\lambda = 0$).

### Recovery Statement 5

For qualitative reachability ($\lambda = 0$) and the heuristic $\mathcal {H} _0$ from Recovery Statement 4, $\mathsf{\mathsf{PrIC3}}$ invokes its core $\mathsf{PrIC3} _{\mathcal {H}}$ exactly *once*.

This statement is true, as $\mathsf{PrIC3} _{\mathcal {H}}$ returns either $\textit{safe}$ or a subsystem containing a path from the initial state to a bad state. In the latter case, $\mathsf{CheckRefutation}$ detects that the subsystem is indeed a counterexample which cannot be spurious in the qualitative setting.

We remark that the procedure $\mathsf{CheckRefutation}$ invoked in l. 5 is a classical fallback; it runs an (alternative) model checking algorithm, e.g., solving the set of Bellman equations, for the subsystem. In the worst case, i.e., for $S' = S$, we thus solve exactly our problem statement. Empirically (Table 1) we observe that for reasonable oracles the procedure $\mathsf{CheckRefutation}$ is invoked on significantly smaller subMDPs. However, in the worst case the subMDP must include *all* paths of the original MDP, and then thus coincides.

**Refine Oracle.** Whenever we have neither proven the MDP safe nor unsafe, we refine the oracle to prevent generating the same subsystem in the next invocation of $\mathsf{PrIC3} _{\mathcal {H}}$. To ensure termination, oracles should only be refined finitely often. That is, we need some progress measure. The set $\textit{touched}$ overapproximates all counterexamples encountered in some invocation of $\mathsf{PrIC3} _{\mathcal {H}}$ and we propose to use its size as the progress measure. While there are several possibilities to update $\textit{touched}$ through the user-defined procedure  (l. 6), every implementation should hence satisfy . Consequently, after finitely many iterations, the oracle is refined with respect to all states. In this case, we may as well rely on solving the characteristic LP problem:

### Lemma 7

The algorithm $\mathsf{\mathsf{PrIC3}}$ in Algorithm 3 is sound and complete if $\mathsf{Refine} (\Omega ,S)$ returns a perfect oracle $\Omega$ (with *S* is the set of all states).

Weaker assumptions on $\mathsf{Refine}$ are possible, but are beyond the scope of this paper. Moreover, the above lemma does not rely on the abstract concept that heuristic $\mathcal {H}$ provides suitable probabilities after finitely many refinements.^8^

## Practical PrIC3

So far, we gave a conceptual view on PrIC3, but now take a more practical stance. We detail important features of effective implementations of PrIC3 (based on our empirical evaluation). We first describe an implementation without generalization, and then provide a prototypical extension that allows for three variants of generalization.

### A Concrete PrIC3 Instance Without Generalization

**Fig. 3.**
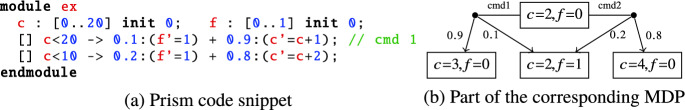
Illustrative Prism-style probabilistic guarded command language example

*Input.* We describe MDPs using the Prism guarded command language^9^, exemplified in Fig. 3. States are described by valuations to *m* (integer-valued) program variables $\text {vars}$, and outgoing actions are described by commands of the formIf a state satisfies guard, then the corresponding action with *k* branches exists; probabilities are given by probi, the successor states are described by updatei, see Fig. 3b.

*Encoding.* We encode frames as logical formulae. Updating frames then corresponds to adding conjuncts, and checking for relative inductivity is a satisfiability call. Our encoding is as follows: States are assignments to the program variables, i.e., $\mathsf {States}= \mathbb {Z}^m$. We use various uninterpreted functions, to whom we give semantics using appropriate constraints. Frames^10^ are represented by uninterpreted functions $\mathsf {Frame} :\mathsf {States} \rightarrow \mathbb {R}$ satisfying $\mathsf {Frame} \left( s \right) = d$ implies $F[s]\ge d$. Likewise, the Bellman operator is an uninterpreted function $\mathsf {Phi} :\mathsf {States} \rightarrow \mathbb {R}$ such that $\mathsf {Phi} \left( s \right) = d$ implies $\Phi \left( F \right) [ s ] \ge d$. Finally, we use $\mathsf {Bad} :\mathsf {States} \rightarrow \mathbb {B}$ with $\mathsf {Bad} \left( s \right)$ iff $s \in B$.

Among the appropriate constraints, we ensure that variables are within their range, bound the values for the frames, and enforce $\mathsf {Phi} \left( s \right) =1$ for $s \in B$. We encode the guarded commands as exemplified by this encoding of the first command in Fig. 3:

$$\begin{aligned}&\forall \,s \in \mathsf {States}:\lnot \mathsf {Bad} \left( s \right) \wedge s[c] < 20 \\ {}&\quad \Longrightarrow \mathsf {Phi} \left( s \right) = 0.1 \cdot \mathsf {Frame} \left( (s[c],1) \right) + 0.9 \cdot \mathsf {Frame} \left( (s[c] + 1,s[f]) \right) . \end{aligned}$$

In our implementation, we optimize the encoding. We avoid the uninterpreted functions by applying an adapted Ackerman reduction. We avoid universal quantifiers, by first observing that we always ask whether a single state is not inductive, and then unfolding the guarded commands in the constraints that describe a frame. That encoding grows linear in the size of the maximal out-degree of the MDP, and is in the quantifier-free fragment of linear arithmetic (QFLRIA).

*Heuristic.* We select probabilities $\delta _i$ by solving the following optimization problem, with variables $x_i$, $\textit{range}(x_i)\in [0,1]$, for states $s_i \in \text {Succs}(s,a)$ and oracle $\Omega$^11^.

$$\begin{aligned}&\text {minimize} \sum ^k_{\begin{array}{c} i \\ s_i \not \in B \end{array}} \left| \frac{x_i}{\sum _{j=1}^k x_j} - \frac{\Omega \left( s_i \right) }{\sum _{j=1}^n \Omega \left( s_j \right) } \right| \nonumber \; \text {s.t.}\; \delta = \sum _{i=1}^k P(s, a , s_i) \cdot {\left\{ \begin{array}{ll} 1, &{} \text {if}~s_i \in B, \\ x_i, &{} \text {else.} \end{array}\right. } \end{aligned}$$

The constraint ensures that, if the values $x_i$ correspond to the actual reachability probabilities from $s_i$, then the reachability from state *s* is exactly $\delta$. A constraint stating that $\delta \ge \ldots$ would also be sound, but we choose equality as it preserves room between the actual probability and the threshold we want to show. Finally, the objective function aims to preserve the ratio between the suggested probabilities.

*Repushing and Breaking Cycles.* *Repushing* [23] is an essential ingredient of both standard IC3 and PrIC3. Intuitively, we avoid opening new frames and spawning obligations that can be deduced from current information. Since repushing generates further obligations in the current frame, its implementation requires that the detection of Zeno-behavior has to be moved from $\mathsf{PrIC3} _{\mathcal {H}}$ into the $\mathsf{Strengthen} _{\mathcal {H}}$ procedure. Therefore, we track the histories of the obligations in the queue. Furthermore, once we detect a cycle we first try to adapt the heuristic $\mathcal {H}$ locally to overcome this cyclic behavior instead of immediately giving up. This local adaption reduces the number of $\mathsf{PrIC3} _{\mathcal {H}}$ invocations.

*Extended Queue.* In contrast to standard IC3, the obligation queue might contain entries that vary only in their $\delta$ entry. In particular, if the MDP is not a tree, it may occur that the queue contains both $(i, s, \delta )$ and $(i, s, \delta ')$ with $\delta > \delta '$. Then, $(i, s, \delta ')$ can be safely pruned from the queue. Similarly, after handling $(i, s, \delta )$, if some fresh obligation $(i, s, \delta '' > \delta )$ is pushed to the queue, it can be substituted with $(i, s, \delta )$. To efficiently operationalize these observations, we keep an additional mapping which remains intact over multiple invocations of $\mathsf{Strengthen} _{\mathcal {H}}$. We furthermore employed some optimizations for $Q.\text {touched}()$ aiming to track potential counterexamples better. After refining the heuristic, one may want to reuse frames or the obligation queue, but empirically this leads to performance degradation as the values in the frames are inconsistent with behavior suggested by the heuristic.

### Concrete PrIC3 with Generalization

So far, frames are updated by changing single entries whenever we resolve obligations $(i, s, \delta )$, i.e., we add conjunctions of the form $F_i[s] \le \delta$. Equivalently, we may add a constraint $\forall s' \in S: F_i[s'] \le p_{\{s\}}(s')$ with $p_{\{s\}}(s) = \delta$ and $p_{\{s\}} = 1$ for all $s' \ne s$.

Generalization in IC3 aims to update a set $G$ (including *s*) of states in a frame rather than a single one without invalidating relative inductivity. In our setting, we thus consider a function $p_{G}:G\rightarrow [0,1]$ with $p_{G}(s) \le \delta$ that assigns (possibly different) probabilities to all states in $G$. Updating a frame then amounts to adding the constraint

$$\begin{aligned} \forall \,s\in \mathsf {States}:s \in G\Longrightarrow \mathsf {Frame} \left( s \right) \le p_G(s). \end{aligned}$$

Standard IC3 generalizes by iteratively “dropping” a variable, say *v*. The set $G$ then consists of all states that do not differ from the fixed state *s* except for the value of *v*.^12^ We take the same approach by iteratively dropping program variables. Hence, $p_{G}$ effectively becomes a mapping from the value *s*[*v*] to a probability. We experimented with four types of functions $p_G$ that we describe for Markov chains. The ideas are briefly outlined below; details are beyond the scope of this paper.

*Constant* $p_G$*.* Setting all $s \in G$ to $\delta$ is straightforward but empirically not helpful.

*Linear Interpolation.* We use a linear function $p_G$ that interpolates two points. The first point $(s[v], \delta )$ is obtained from the obligation $(i,s,\delta )$. For a second point, consider the following: Let $\text {Com}$ be the unique^13^ command active at state *s*. Among all states in $G$ that are enabled in the guard of $\text {Com}$, we take the state $s'$ in which $s'[v]$ is maximal^14^. The second point for interpolation is then $(s'[v], \Phi \left( F_{i-1} \right) [s'])$. If the relative inductivity fails for $p_G$ we do not generalize with $p_G$, but may attempt to find other functions.

*Polynomial Interpolation.* Rather than linearly interpolating between two points, we may interpolate using more than two points. In order to properly fit these points, we can use a higher-degree polynomial. We select these points using counterexamples to generalization (CTGs): We start as above with linear interpolation. However, if $p_G$ is not relative inductive, the SMT solver yields a model with state $s'' \in G$ and probability $\delta ''$, with $s''$ violating relative inductivity, i.e., $\Phi \left( F_{i-1} \right) [s''] > \delta ''$. We call $(s'', \Phi \left( F_{i-1} \right) [s''])$ a CTG, and $(s''[v],\Phi \left( F_{i-1} \right) [s'']))$ is then a further interpolation point, and we repeat.

Technically, when generalizing using nonlinear constraints, we use real-valued arithmetic with a branch-and-bound-style approach to ensure integer values.

*Hybrid Interpolation.* In polynomial interpolation, we generate high-degree polynomials and add them to the encoding of the frame. In subsequent invocations, reasoning efficiency is drastically harmed by these high-degree polynomials. Instead, we soundly approximate $p_G$ by a piecewise linear function, and use these constraints in the frame.

## Experiments

We assess how PrIC3 may contribute to the state of the art in probabilistic model checking. We do some early empirical evaluation showing that PrIC3 is feasible. We see ample room for further improvements of the prototype.

*Implementation.* We implemented a prototype^15^ of PrIC3 based on Sect. 6.1 in Python. The input is represented using efficient data structures provided by the model checker Storm. We use an incremental instance of Z3 [47] for each frame, as suggested in [23]. A solver for each frame is important to reduce the time spent on pushing the large frame-encodings. The optimization problem in the heuristic is also solved using Z3. All previously discussed generalizations (none, linear, polynomial, hybrid) are supported.

*Oracle and Refinement.* We support the (pre)computation of four different types of oracles for the  step in Algorithm 3: (1) A perfect oracle solving *exactly* the Bellman equations. Such an oracle is unrealistic, but interesting from a conceptual point. (2) Relative frequencies by recording all visited states during simulation. This idea is a naïve simplification of Q-learning. (3) Model checking with decision diagrams (DDs) and few value iterations. Often, a DD representation of a model can be computed fast, and the challenge is in executing sufficient value iterations. We investigate whether doing few value iterations yields a valuable oracle (and covers states close to bad states). (4) Solving a (pessimistic) LP from BFS partial exploration. States that are not expanded are assumed bad. Roughly, this yields oracles covering states close to the initial states.

To implement  (cf. Algorithm 3, l. 7), we create an LP for the subMDP induced by the touched states. For states whose successors are not in the touched states, we add a transition to *B* labeled with the oracle value as probability. The solution of the resulting LP updates the entries corresponding to the touched states.

For  (cf. Algorithm 3, l. 6), we take the union of the subsystem and the touched states. If this does not change the set of touched states, we also add its successors.

*Setup.* We evaluate the run time and memory consumption of our prototype of PrIC3. We choose a combination of models from the literature (BRP [21], ZeroConf [18]) and some structurally straightforward variants of grids (chain, double chain; see [11, Appendix A]). Since our prototype lacks the sophisticated preprocessing applied by many state-of-the-art model checkers, it is more sensitive to the precise encoding of a model, e.g., the number of commands. To account for this, we generated new encodings for all models. All experiments were conducted on an single core of an Intel® Xeon® Platinum 8160 processor. We use a 15 min time-limit and report TO otherwise. Memory is limited to 8GB; we report MO if it is exceeded. Apart from the oracle, all parameters of our prototype remain fixed over all experiments. To give an impression of the run times, we compare our prototype with both the explicit (Storm$_\text {sparse}$) and DD-based (Storm$_\text {dd}$) engine of the model checker Storm 1.4, which compared favourably in QComp [29].

*Results.* In Table 1, we present the run times for various invocations of our prototype and Oracle 4^16^. In particular, we give the model name and the number of (non-trivial) states in the particular instance, and the (estimated) actual probability to reach *B*. For each model, we consider multiple thresholds $\lambda$. The next 8 columns report on the four variants of PrIC3 with varying generalization schemes. Besides the scheme with the run times, we report for each scheme the number of states of the largest (last) subsystem that $\mathsf{CheckRefutation}$ in Algorithm 3, l. 5 was invoked upon (column |*sub*|). The last two columns report on the run times for Storm that we provide for comparison. In each row, we mark with  MDPs that are unsafe, i.e., PrIC3 refutes these MDPs for the given threshold $\lambda$. We **highlight** the best configurations of PrIC3.

**Table 1.** Empirical results. Run times are in seconds; time out = 15 min.

	|*S*|	$\text {Pr}^{\text {max}} \left( s_I \models \lozenge B \right)$	$\lambda$	w/o	$|{\textit{sub}}|$	lin	$|{\textit{sub}}|$	pol	$|{\textit{sub}}|$	hyb	$|{\textit{sub}}|$	Storm$_\text {sparse}$	Storm$_\text {dd}$
BRP	$10^3$	0.035	0.1	TO	–	TO	–	TO	–	TO	–	${<}0.1$	0.12
				$\mathbf {51.3}$	324	125.8	324	TO	–	MO	–	${<}0.1$	0.18
				$\mathbf {10.9}$	188	38.3	188	TO	–	MO	–	${<}0.1$	0.1
ZeroConf	$10 ^{4}$	0.5	0.9	TO	–	TO	–	0.4	0	$\mathbf {0.1}$	0	${<}0.1$	296.8
			0.52	TO	–	TO	–	0.2	0	$\mathbf {0.2}$	0	${<}0.1$	282.6
				$\mathbf {{<}0.1}$	1	$\mathbf {{<}0.1}$	1	$\mathbf {{<}0.1}$	1	$\mathbf {{<}0.1}$	1	${<}0.1$	300.2
	$10^9$	${\sim }0.55$	0.9	TO	–	TO	–	$\mathbf {3.7}$	0	MO	–	MO	TO
			0.75	TO	–	TO	–	$\mathbf {3.4}$	0	MO	–	MO	TO
			0.52	TO	–	TO	–	TO	–	TO	–	MO	TO
				$\mathbf {{<}0.1}$	1	$\mathbf {{<}0.1}$	1	$\mathbf {{<}0.1}$	1	$\mathbf {{<}0.1}$	1	MO	TO
Chain	$$ 10 ^3$$	0.394	0.9	18.8	0	60.2	0	1.2	0	$$\mathbf {0.3}$$	0	$${<}0.1$$	$${<}0.1$$
			0.4	20.1	0	55.4	0	$\mathbf {0.9}$	0	TO	–	${<}0.1$	${<}0.1$
				$\mathbf {91.8}$	431	119.5	431	TO	–	TO	–	${<}0.1$	${<}0.1$
				$\mathbf {46.1}$	357	64.0	357	TO	–	TO	–	${<}0.1$	${<}0.1$
	$10 ^4$	0.394	0.9	TO	–	TO	–	1.6	0	$\mathbf {0.3}$	0	${<}0.1$	4.5
			0.4	TO	–	TO	–	$\mathbf {1.4}$	0	TO	–	${<}0.1$	4.9
				TO	–	TO	–	TO	–	TO	–	${<}0.1$	4.9
	$10 ^{12}$	0.394	0.9	TO	–	TO	–	$\mathbf {6.4}$	0	MO	–	MO	TO
			0.4	TO	–	TO	–	$\mathbf {6.0}$	0	MO	–	MO	TO
Double chain	$$ 10 ^3$$	0.215	0.9	528.1	0	828.8	0	203.3	0	$$\mathbf {0.6}$$	0	$${<}0.1$$	$${<}0.1$$
			0.3	588.4	0	TO	–	138.3	0	$\mathbf {0.5}$	0	${<}0.1$	${<}0.1$
			0.216	$\mathbf {597.4}$	0	TO	–	765.8	0	MO	–	${<}0.1$	${<}0.1$
				TO	–	TO	–	TO	–	TO	–	${<}0.1$	${<}0.1$
	$10 ^{4}$	0.22	0.3	TO	–	TO	–	17.5	0	$\mathbf {0.5}$	0	0.2	2.6
			0.24	TO	–	TO	–	$\mathbf {16.8}$	0	MO	–	0.2	2.7
	$10^7$	$2.6\mathbf {E}^{-4}$	$4\mathbf {E}^{-3}$	TO	–	TO	–	TO	–	MO	–	TO	TO
			$2.7\mathbf {E}^{-4}$	TO	–	TO	–	$\mathbf {281.2}$	0	MO	–	TO	TO

*Discussion.* Our experiments give a mixed picture on the performance of our implementation of PrIC3. On the one hand, Storm significantly outperforms PrIC3 on most models. On the other hand, PrIC3 is capable of reasoning about huge, yet simple, models with up to $10^{12}$ states that Storm is unable to analyze within the time and memory limits. There is more empirical evidence that PrIC3 may complement the state-of-the-art:

First, *the size of thresholds matters*. Our benchmarks show that—at least without generalization—more “wiggle room” between the precise maximal reachability probability and the threshold generally leads to a better performance. PrIC3 may thus prove bounds for large models where a precise quantitative reachability analysis is out of scope.

Second, PrIC3*enjoys the benefits of bounded model checking*. In some cases, e.g., ZeroConf for $\lambda = 0.45$, PrIC3 refutes very fast as it does not need to build the whole model.

Third, if PrIC3 proves the safety of the system, it does so without relying on checking large subsystems in the $\mathsf{CheckRefutation}$ step.

Fourth, *generalization is crucial*. Without generalization, PrIC3 is unable to prove safety for any of the considered models with more than $10^3$ states. With generalization, however, it can prove safety for very large systems and thresholds close to the exact reachability probability. For example, it proved safety of the Chain benchmark with $10^{12}$ states for a threshold of 0.4 which differs from the exact reachability probability by 0.006.

Fifth, *there is no best generalization*. There is no clear winner out of the considered generalization approaches. Linear generalization always performs worse than the other ones. In fact, it performs worse than no generalization at all. The hybrid approach, however, occasionally has the edge over the polynomial approach. This indicates that more research is required to find suitable generalizations.

In [11, Appendix A], we also compare the additional three types of oracles (1–3). We observed that only few oracle refinements are needed to prove *safety*; for small models at most one refinement was sufficient. However, this does not hold if the given MDP is unsafe. DoubleChain with $\lambda = 0.15$, for example, and Oracle 2 requires 25 refinements.

## Conclusion

We have presented PrIC3—the first truly probabilistic, yet conservative, extension of IC3 to quantitative reachability in MDPs. Our theoretical development is accompanied by a prototypical implementation and experiments. We believe there is ample space for improvements including an in-depth investigation of suitable oracles and generalizations.

## Footnotes

1 Recently, (standard) IC3 for TSs was *utilized* in model checking Markov chains [49] to on-the-fly compute the states that cannot reach *B*.

2 In [38], tree-like counterexamples are used for non-linear predicate transformers in IC3.

3 Maximal with respect to all possible resolutions of nondeterminism in the MDP.

4 In IC3, frames are typically characterized by logical formulae. To understand IC3 ’s fundamental principle, however, we prefer to think of frames as functions in $\{0,1\}^{S}$ partially ordered by $\le$.

5 We assume that the set $\text {Succs}(s,a) = \left\{ s' \in S ~\mid ~ P(s, a , s') > 0 \right\}$ of *relevant a-successors* of state *s* is returned in some arbitrary, but fixed order.

6 $Q.\text {touched}()$ might be restricted to only contain this path by some simple adaptions.

7 We thus assume that heuristic $\mathcal {H}$ invokes the oracle whenever it needs to guess some probability.

8 One could of course now also create a heuristic that is trivial for a perfect oracle and invoke $\mathsf{PrIC3} _{\mathcal {H}}$ with the heuristic for the perfect oracle, but there really is no benefit in doing so.

9 Preprocessing ensures a single thread (module) and no deadlocks.

10 In each operation, we only consider a single frame.

11 If $\max \Omega (s_j) = 0$, we assume $\forall j. \Omega (s_j) = 0.5$. If $\delta = 0$, we omit rescaling to allow $\sum x_j =0$.

12 Formally, $$G= \{ s' \mid \text {for all } v' \in \text {vars}\setminus \{ v \}: s'(v') = s(v')\}$$.

13 Recall that we have a Markov chain consisting of a single module.

14 This implicitly assumes that *v* is increased. Adaptions are possible.

15 The prototype is available open-source from https://github.com/moves-rwth/PrIC3.

16 We explore $\min \{|S|, 5000\}$ states using BFS and Storm.

## References

[1] Ábrahám E, Becker B, Dehnert C, Jansen N, Katoen J-P, Wimmer R, Bernardo M, Damiani F, Hähnle R, Johnsen EB, Schaefer I (2014). Counterexample generation for discrete-time Markov models: an introductory survey. Formal Methods for Executable Software Models.

[2] Agha G, Palmskog K (2018). A survey of statistical model checking. ACM Trans. Model. Comput. Simul..

[3] Agrawal, S., Chatterjee, K., Novotný, P.: Lexicographic ranking supermartingales: an efficient approach to termination of probabilistic programs. In: PACMPL 2(POPL), pp. 34:1–34:32 (2018)

[4] de Alfaro L, Kwiatkowska M, Norman G, Parker D, Segala R, Graf S, Schwartzbach M (2000). Symbolic model checking of probabilistic processes using MTBDDs and the kronecker representation. Tools and Algorithms for the Construction and Analysis of Systems.

[5] Baier C, de Alfaro L, Forejt V, Kwiatkowska M (2018). Model checking probabilistic systems. Handbook of Model Checking.

[6] Baier C, Hermanns H, Katoen J-P, Steffen B, Woeginger G (2019). The 10,000 facets of MDP model checking. Computing and Software Science.

[7] Baier C, Katoen J-P (2008). Principles of Model Checking.

[8] Baier C, Klein J, Leuschner L, Parker D, Wunderlich S, Majumdar R, Kunčak V (2017). Ensuring the reliability of your model checker: interval iteration for markov decision processes. Computer Aided Verification.

[9] Barthe G, Espitau T, Ferrer Fioriti LM, Hsu J, Chaudhuri S, Farzan A (2016). Synthesizing probabilistic invariants via Doob’s decomposition. Computer Aided Verification.

[10] Bartocci E, Kovács L, Stankovič M, Chen Y-F, Cheng C-H, Esparza J (2019). Automatic generation of moment-based invariants for prob-solvable loops. Automated Technology for Verification and Analysis.

[11] Batz, K., Junges, S., Kaminski, B.L., Katoen, J.-P., Matheja, C., Schröer, P.: Pric3: Property directed reachability for MDPS. ArXiv e-prints (2020). https://arxiv.org/abs/2004.14835

[12] Biere, A.: Bounded model checking, Handbook of Satisfiability. Frontiers in Artificial Intelligence and Applications, vol. 185, pp. 457–481. IOS Press (2009)

[13] Bradley AR, Jhala R, Schmidt D (2011). SAT-based model checking without unrolling. Verification, Model Checking, and Abstract Interpretation.

[14] Brázdil T, Chatterjee K, Chmelík M, Forejt V, Křetínský J, Kwiatkowska M, Parker D, Ujma M, Cassez F, Raskin J-F (2014). Verification of Markov decision processes using learning algorithms. Automated Technology for Verification and Analysis.

[15] Chadha R, Viswanathan M (2010). A counterexample-guided abstraction-refinement framework for Markov decision processes. ACM Trans. Comput. Logist..

[16] Chakarov A, Sankaranarayanan S, Sharygina N, Veith H (2013). Probabilistic program analysis with martingales. Computer Aided Verification.

[17] Chakraborty, S., Fried, D., Meel, K.S., Vardi, M.Y.: From weighted to unweighted model counting. In: IJCAI, pp. 689–695. AAAI Press (2015)

[18] Cheshire S, Aboba B, Guttman E (2005). Dynamic configuration of ipv4 link-local addresses. RFC.

[19] Cimatti A, Griggio A, Mover S, Tonetta S (2016). Infinite-state invariant checking with IC3 and predicate abstraction. FMSD.

[20] D’Argenio, P.R., Hartmanns, A., Sedwards, S.: Lightweight statistical model checking in nondeterministic continuous time. In: Margaria, T., Steffen, B. (eds.) ISoLA 2018. LNCS, vol. 11245, pp. 336–353. Springer, Cham (2018). 10.1007/978-3-030-03421-4_22

[21] D’Argenio, P.R., Jeannet, B., Jensen, H.E., Larsen, K.G.: Reachability analysis of probabilistic systems by successive refinements. In: de Alfaro, L., Gilmore, S. (eds.) PAPM-PROBMIV 2001. LNCS, vol. 2165, pp. 39–56. Springer, Heidelberg (2001). 10.1007/3-540-44804-7_3

[22] Dehnert C, Junges S, Katoen J-P, Volk M, Majumdar R, Kunčak V (2017). A Storm is coming: a modern probabilistic model checker. Computer Aided Verification.

[23] Eén, N., Mishchenko, A., Brayton, R.K.: Efficient implementation of property directed reachability. In: FMCAD, pp. 125–134. FMCAD Inc. (2011)

[24] Fränzle M, Hermanns H, Teige T, Egerstedt M, Mishra B (2008). Stochastic satisfiability modulo theory: a novel technique for the analysis of probabilistic hybrid systems. Hybrid Systems: Computation and Control.

[25] Gretz F, Katoen J-P, McIver A, Joshi K, Siegle M, Stoelinga M, D’Argenio PR (2013). Prinsys—On a Quest for Probabilistic Loop Invariants. Quantitative Evaluation of Systems.

[26] Gretz F, Katoen J-P, McIver A (2014). Operational versus weakest pre-expectation semantics for the probabilistic guarded command language. Perform. Eval..

[27] Gurfinkel, A., Ivrii, A.: Pushing to the top. In: FMCAD, pp. 65–72. IEEE (2015)

[28] Haddad S, Monmege B (2018). Interval iteration algorithm for MDPs and IMDPs. Theor. Comput. Sci..

[29] Hahn EM, Hartmanns A, Hensel C, Klauck M, Klein J, Křetínský J, Parker D, Quatmann T, Ruijters E, Steinmetz M, Beyer D, Huisman M, Kordon F, Steffen B (2019). The 2019 comparison of tools for the analysis of quantitative formal models. Tools and Algorithms for the Construction and Analysis of Systems.

[30] Hahn EM, Hermanns H, Wachter B, Zhang L, Esparza J, Majumdar R (2010). PASS: abstraction refinement for infinite probabilistic models. Tools and Algorithms for the Construction and Analysis of Systems.

[31] Hahn EM, Li Y, Schewe S, Turrini A, Zhang L, Jones C, Pihlajasaari P, Sun J (2014). iscasMc: a web-based probabilistic model checker. FM 2014: Formal Methods.

[32] Han T, Katoen J-P, Damman B (2009). Counterexample generation in probabilistic model checking. IEEE Trans. Software Eng..

[33] Hark, M., Kaminski, B.L., Giesl, J., Katoen, J.-P.: Aiming low is harder: Induction for lower bounds in probabilistic program verification. In: PACMPL 4(POPL), 37:1–37:28 (2020)

[34] Hartmanns A, Hermanns H, Ábrahám E, Havelund K (2014). The modest toolset: an integrated environment for quantitative modelling and verification. Tools and Algorithms for the Construction and Analysis of Systems.

[35] Hartmanns, A., Kaminski, B.L.: Optimistic value iteration. CAV. LNCS, Springer (2020). [to appear]

[36] Hassan, Z., Bradley, A.R., Somenzi, F.: Better generalization in IC3. In: FMCAD, pp. 157–164. IEEE (2013)

[37] Hermanns H, Wachter B, Zhang L, Gupta A, Malik S (2008). Probabilistic CEGAR. Computer Aided Verification.

[38] Hoder K, Bjørner N, Cimatti A, Sebastiani R (2012). Generalized property directed reachability. Theory and Applications of Satisfiability Testing – SAT 2012.

[39] Kaminski, B.L.: Advanced Weakest Precondition Calculi for Probabilistic Programs. Ph.D. thesis, RWTH Aachen University, Germany (2019). http://publications.rwth-aachen.de/record/755408/files/755408.pdf

[40] Kaminski BL, Katoen J-P, Matheja C, Olmedo F (2018). Weakest precondition reasoning for expected runtimes of randomized algorithms. J. ACM.

[41] Kattenbelt M, Kwiatkowska MZ, Norman G, Parker D (2010). A game-based abstraction-refinement framework for Markov decision processes. FMSD.

[42] Kozen, D.: A probabilistic PDL. In: STOC, pp. 291–297. ACM (1983)

[43] Kwiatkowska M, Norman G, Parker D, Gopalakrishnan G, Qadeer S (2011). PRISM 4.0: verification of probabilistic real-time systems. Computer Aided Verification.

[44] Lange, T., Neuhäußer, M.R., Noll, T., Katoen, J.-P.: IC3 software model checking. In: STTT, vol. 22, pp. 135–161 (2020)

[45] Lassez JL, Nguyen VL, Sonenberg L (1982). Fixed point theorems and semantics: a folk tale. Inf. Process. Lett..

[46] McIver A, Morgan C (2005). Abstraction, Refinement and Proof for Probabilistic Systems.

[47] de Moura L, Bjørner N, Ramakrishnan CR, Rehof J (2008). Z3: an efficient SMT solver. Tools and Algorithms for the Construction and Analysis of Systems.

[48] Park D (1969). Fixpoint induction and proofs of program properties. Machine intelligence.

[49] Polgreen, E., Brain, M., Fränzle, M., Abate, A.: Verifying reachability properties in Markov chains via incremental induction. CoRR abs/1909.08017 (2019)

[50] Puterman, M.L.: Markov Decision Processes: Discrete Stochastic Dynamic Programming. Wiley Series in Probability and Statistics, Wiley, Hoboken (1994)

[51] Quatmann T, Katoen J-P, Chockler H, Weissenbacher G (2018). Sound value iteration. Computer Aided Verification.

[52] Rabe MN, Wintersteiger CM, Kugler H, Yordanov B, Hamadi Y, Norman G, Sanders W (2014). Symbolic approximation of the bounded reachability probability in large Markov chains. Quantitative Evaluation of Systems.

[53] Seufert, T., Scholl, C.: Sequential verification using reverse PDR. MBMV. pp. 79–90. Shaker Verlag (2017)

[54] Suenaga K, Ishizawa T, Beyer D, Zufferey D (2020). Generalized property-directed reachability for hybrid systems. Verification, Model Checking, and Abstract Interpretation.

[55] Takisaka T, Oyabu Y, Urabe N, Hasuo I, Lahiri SK, Wang C (2018). Ranking and repulsing supermartingales for reachability in probabilistic programs. Automated Technology for Verification and Analysis.

[56] Vazquez-Chanlatte, M., Rabe, M.N., Seshia, S.A.: A model counter’s guide to probabilistic systems. CoRR abs/1903.09354 (2019)

[57] Wimmer R, Braitling B, Becker B, Jones ND, Müller-Olm M (2008). Counterexample generation for discrete-time markov chains using bounded model checking. Verification, Model Checking, and Abstract Interpretation.

